# A Study of Couple Burnout in Infertile Couples

**DOI:** 10.5539/gjhs.v8n4p158

**Published:** 2015-08-06

**Authors:** Fatemeh Ghavi, Safieh Jamale, Leili Mosalanejad, Zahra Mosallanezhad

**Affiliations:** 1 Faculty of Nursing and Midwifery, Shiraz University of Medical Sciences, Shiraz, Iran; 2 Midwifery Department, Nursing School, Research Center for Social Determinants of Health, Jahrom University of Medical Sciences, Jahrom, Iran; 3 Mental Health Department, Research Center for Social Determinants of Health, Jahrom University of Medical Sciences, Jahrom, Iran; 4 Student Research Committee, Department of OB-GYN, School of Medicine, Shiraz University of Medical Sciences, Shiraz, Iran

**Keywords:** burnout, couples, infertility

## Abstract

**Introduction:**

Infertility is a major crisis that can cause psychological problems and emotionally distressing experiences, and eventually affect a couples’ relationship. The objective of this study is to investigate couple burnout in infertile couples who were undergoing treatmentat the Infertility Clinic of Yazd, Iran.

**Method:**

The present study is a cross-sectional descriptive one on 98 infertile couples referringto the Infertility Centerof Yazd, Iran, who were chosen on a simple random sampling basis. The measuring tools consisted of the Couple Burnout Measure (CBM) and a demographic questionnaire. The collected data were analyzed using SPSS 16 and the statistical tests of ANOVA and t-test. P-values less than 0.05 were considered as significant.

**Results:**

The results show that infertile women experience higher levels of couple burnout than their husbands (p<0.001). Also, a comparison of the scales of couple burnout—psychological burnout (p<0.01), somatic burnout (p<0.01), and emotional burnout (p<0.001)—between wives and husbands show that women are at greater risk.

**Conclusion:**

Infertile couples’ emotional, mental, and sexual problems need to be addressed as part of the infertility treatment programs, and psychotherapists should be included in the medical team.

## 1. Introduction

Infertility is defined as non-pregnancy after having unprotected sex for one year. According to WHO, the global rate of infertility is approximately between 10% to 15%. In Iran, however, the rate of infertility is reported to be above 20% (Berek, 2011). Though prevalence of infertility varies considerably between countries, WHO recognizesinfertility as a public health issue that can threaten individuals, families and societies across the globe (Boivin et al., 2007; Burns & Covington, 2006). Moreover, infertility is described as a stress factor that can turn into a crisis in a couple’s life, reduce their quality of life, and cause marital conflicts (Chachamovich et al., 2009; Klemetti et al., 2010). Studies show that infertility can lead to psychological disorders, including couple burnout and sexual dysfunction (Anderson et al., 2003). In a multicenter study of 604 infertile women in Iran, the results showed that 56% of the subjects were suffering from sexual dysfunction (Pakpour et al., 2012).

Sexual satisfaction is adversely affected by such consequences of infertility as poor self-confidence, depression, and burnout (Mechanickbraverman, 2004). Burnout is a state of physical, psychological, and emotional exhaustion, caused by incompatibility between expectations and reality. Burnout is accompanied by such symptoms as physical exhaustion, listlessness and indifference. Emotional exhaustion is characterized by feelings of resentment, unwillingness to resolve problems, despair, depression, meaninglessness, and even suicidal tendencies. Symptoms of psychological exhaustion include low self-esteem, a negative view of one’s spouse, dissatisfaction with one’s spouse, and dissatisfaction with and disliking oneself (Ayala Malach, 2003). Such factors as illogical ideas, unrealistic expectations, and hardships of life can contribute to sexual burnoutin varying degrees, depending on the couple’s compatibility and beliefs (Bernstein & Bernstein, 2002).

The results of a study by Nikoobakht et al. show that there is a positive correlation between couple burnout and infertility (Nikoubakht et al., 2012). According to a study on infertile couples in South Africa, 43% of infertile women believe that their inability to have a child has serious negative effects on their lives, especially their sexual relationships (Lee et al., 2001). Most men and women believe that their masculinity or femininity suffers as a result of their infertility; this view, in turn, adversely affects their sexual satisfaction (Mechanickbraverman, 2004).

A large number of studies have addressed sexual dysfunction in infertile women; however, few studies have addressed couple burnout in infertile couples, either in Iran or abroad. In view of the importance of a healthy sexual relationshipin marital satisfaction and the well-being of the family, this study aimed at exploring couple burnout in infertile couples.

## 2. Materials and Methods

This is a cross-sectional study. The sample, which was chosen using convenience sampling, consisted of 92 infertile couples who were visitingthe Infertility Center of Yazd, Iran. The criteria for including the infertile women and their husbands were being Iranian and speaking Farsi. The criteria for excluding couples were suffering from a disabling mental or physical disease and taking sexual suppressant medicine, e.g.anti-hypertensive medications, digoxin, and anti-depression medications. Initially, every couple was briefly informed about the objectives of the study, how to complete the questionnaire, and freedom to participate in the study.

To collect data, the researchers used a two-part questionnaire: part one dealt with demographic characteristics, i.e., age, sex, length of infertility, occupation, education, and length of marriage; part two was the Couple Burnout Measure (CBM).

CBM is a self-assessment tool designed to measure the extent of couple burnout in couples (Pines, 1996). The questionnaire consists of 21 items which fall into three categories: somatic burnout (fatigue, lethargy, and sleep disorders), emotional burnout (depression, despair, and feeling trapped), and psychological burnout (feelings of worthlessness, frustration, and anger toward one’s spouse). The entire items are scored on a seven-point scale: scale 1 indicates not having experienced the situation referred to at all, and scale 7 indicates having experienced the situation frequently. An evaluation of the reliability of CBM showed that the internal homogeneity of the items ranged from 0.84 to 0.90. The validity of the questionnaire was verified by negative correlations with positive relationship qualities, e.g. having a positive view of the relationship, having quality conversations with one’s spouse, safety, self-improvement, having a sense of purpose, being emotionally attracted to one’s spouse, and the quality of the sexual relationship. In Iran, Navidi used the questionnaire with 240 subjects (120 nurses and 120 teachers) and found the Chronbach’s alpha of the questionnaire to be 0.86 (Navid, 2006). In Adibrad’s study, the test-retest reliability coefficientof the questionnaire was found to be 0.89after a one-month interval, 0.76 after a two-month interval, and 0.66 after a four-month interval. Alpha coefficient, which was used to assess the internal consistency, was found to be between 0.91 and 0.93 for most of the participants (Adibrad & Adibrad, 2005).

## 3. Results

Table 1 shows that somatic, emotional, and psychological burnouts in particular and sexual burnout in general were greater in women than in men.

**Table 1:** Differences between the two groups’ sexual burnout mean scores

	Sex	N	Mean	SD	T*	p
**Somatic**	Female	92	24.03	4.65	2.603	0.01
	male	76	2.27	3.9		
**Emotional**	Female	91	19.61	8.90	3.93	0.001
	male	85	15.08	5.99		
**Psychological**	Female	95	27.05	8.67	2.44	0.01
	male	92	24.20	7.11		
**Burnout**	Female	95	72.48	11.30	4.93	0.001
	male	66	62.72	13.68		

* Analysis based on student t- test.

Table 2 shows that the absence of such demographic factors as occupation, length of infertility, education, length of marriage and age, sexual burnout in both sexes has significant values.

**Table 2:** Comparison of the differences incouple burnout by gender

Dependent variable	Source	Sum of squares	DF	Ms	F*	P
**Somatic**	Sex	115.76	1	115.79	9.84	0.002
	Error	6183.77	95	65.09		
**Emotional**	Sex	127.52	1	127.52	1.95	0.16
	Error	3844.67	103	37.32		
**Psychological**	Sex	77.88	1	77.88	2.08	0.15
	Error	1070.48	91	11.76		
**Burnout**	Sex	3698.03	1	3698.03	29.60	0.0001
	Error	9119.98	73	124.93		

* Analysis of Covariance (ANCOVA).

**Figure 1:**
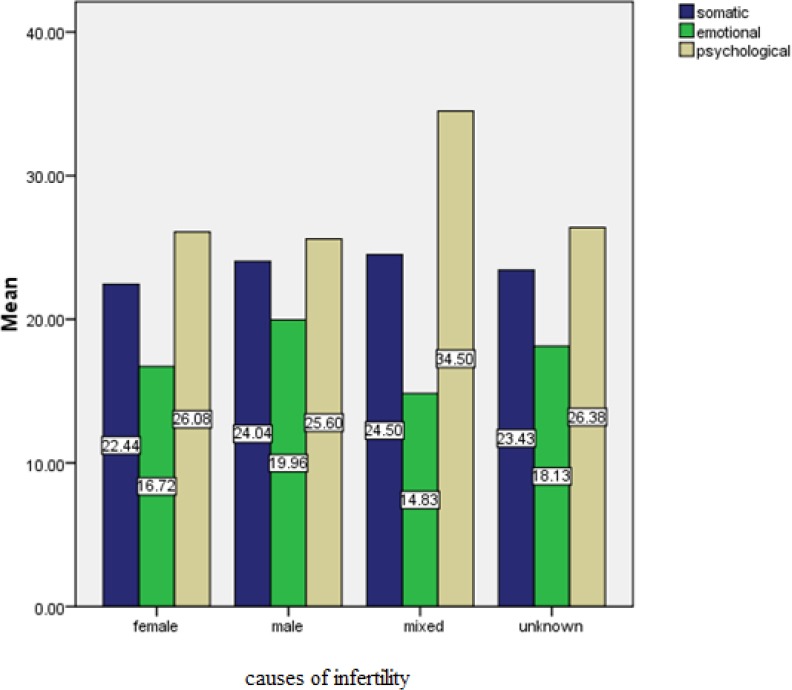
Couple Burnout based on cause of infertilityin couples

Figure 1 shows that in both sexes, the extent of somatic burnout and psychological burnout is related to mixed causes, and the extent of emotional burnout is related to masculine causes.

**Figure 2:**
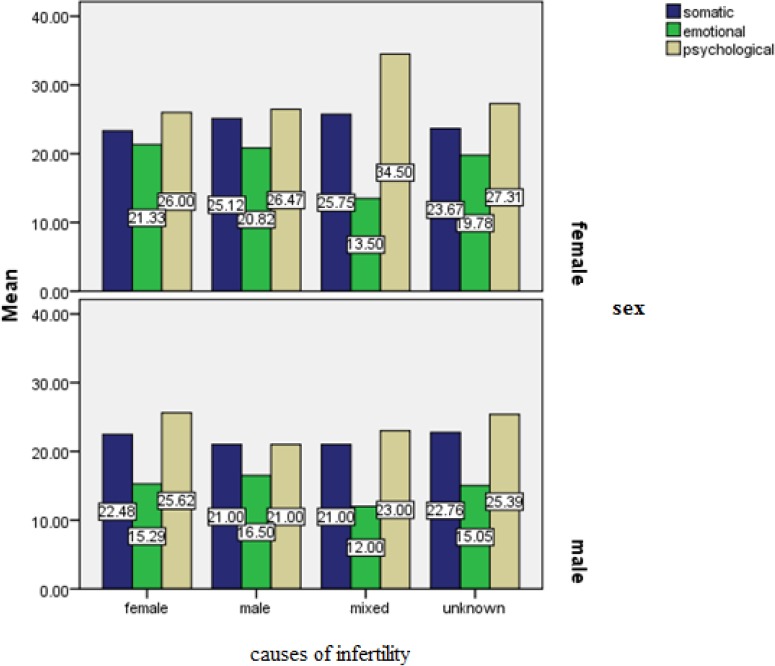
Comparison of burnout based on cause of infertilityby gender

Figure 2 shows that, depending on the reason for infertility, in most women somatic burnout is related to masculine, feminine, or mixed reasons, emotional burnout is related to feminine reasons, and psychological burnout is related to feminine-masculine reasons. In most men, somatic burnout is related to unknown reasons, emotional burnout is related to masculine reasons, and psychological burnout is related to feminine reasons.

**Figure 3:**
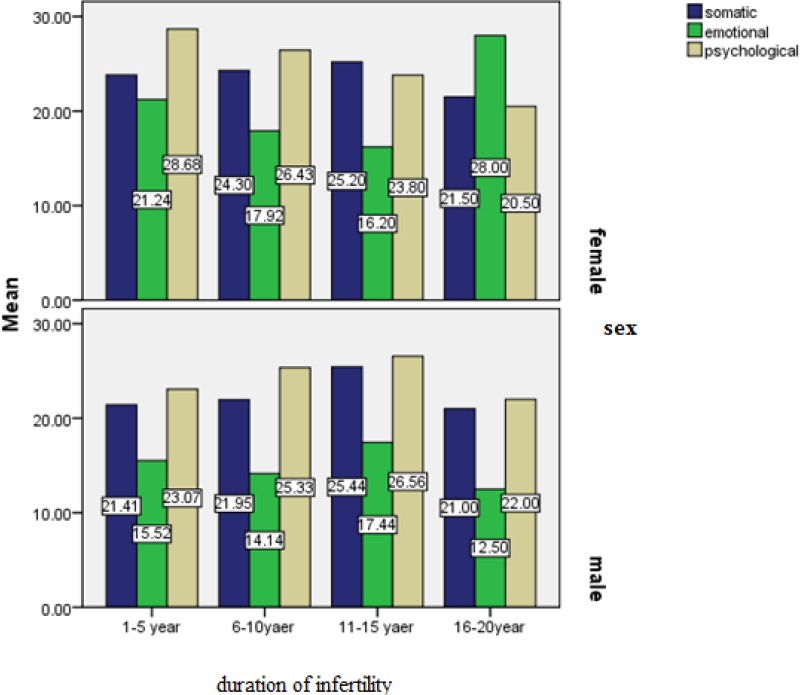
Couple Burnout based on duration of infertility

Figure 3 shows that, depending on the duration of infertility, in most women somatic burnout is related to 11-15 years of infertility, emotional burnout is related to 16-20 yearsof infertility, and psychological burnout is related to 1-5 years of infertility. In most men, all types of burnout (somatic, emotional, and psychological)are related to 11-15 years of infertility. It can be concluded that in both men and women, length of infertility correlates with the degree of burnout.

## 4. Discussion

This study aimed at exploring couple burnout in infertile couples. The analysis of couple burnout in infertile couples shows that infertile women experience higher levels of burnout than their husbands. Moreover, a comparison of the mean scores of couple burnout scales (somatic burnout, psychological burnout, and emotional burnout) shows that infertile women experience significantly greater burnout than their husbands.

It was found that sexual dysfunctions are more common in infertile women. Infertility-related stress in women has a significantly greater impact on their sexuality than other sources of stress. Sexual dysfunctions can, by reducing the frequency of intercourses, cause infertility (Millheiser et al., 2010). In Wischmann’s review study, it is stated that the impact of infertility on women’s sexual lives is significantly greater than on men’s (Wischmann, 2013). The results of a study of 300 infertile couples in Taiwan showed that infertile women experienced more burnout and sexual dissatisfaction than their husbands (Lee et al., 2001). These results are in agreementwith the results of the present study about the impact of infertility on somatic, emotional and psychological burnout. In a study in Turkey, 44% of women, compared to 20% of men, experienced loss of sexual desire following the diagnosis of their infertility (Tuzer et al., 2010). Other studies also show thatdiagnosis of infertility affects women’s sexual motivation and desire more than men’s (Marci et al., 2012).

Similarly, Lee et al. conclude that women experience less sexual satisfaction than their husbands in all types of infertility, except those where the cause is unknown (Lee et al., 2001). Thesefindings confirm the adverse effect of psychological burnout caused by infertility on a couple’s marital satisfaction. The present study shows that in both sexes, sexual burnout has a significant impact on both general burnout and somatic burnout. Somatic burnout may affect other aspects of a couple’s life and may lead tocertain psychiatric problems. The present study shows that the significant differences between general and somatic burnout are present in both sexes. The higher rate of somatic and physical burnout in women can adversely affect the entire aspects of a couple’s life and lead to psychological disorders. It is also associated with marital issues and dissatisfaction in infertile couples.

In their study of the impact of infertility on couples’ quality of life, marital adjustment, andsexual function, Mongm et al. maintain that infertile women are significantly less successful at psychological and marital adjustment; fertile and infertile men, however, did not differ in terms of psychological and marital adjustment; however, infertile women’s psychological well-being and quality of life were influenced more than infertile men’s (Mongm et al., 2004).

Studies show that women are more vulnerable to the adverse effects of infertility on psychological health than men, and confirm the overall contribution of infertility to couple burnout. Many studies conclude that infertile women experience higher levels of stress, anxiety and depression than men, which in turn results in marital dissatisfaction and poor quality of life and mental health (Mongm et al., 2004; Andrews et al. 1991, 1992; Berg & Wilson, 1990).

One of the possible reasons for women’s higher couple burnout scores compared to their husbandsis women’s great respect for values in most cultures, especially the Iranian culture. Since having children is traditionally recognized as a woman’s primary role (Bagshawe & Taylor, 2003), the negative psychological effects of infertility affect women more than men. While many infertile women state that a childless life is inconceivable to them, men express feelings of a different nature (Khayata et al., 2003). Another possible reason is the treatment methods for infertility which can affect a couple’s sexual relationship and, by creating a sense ofnecessity and inevitability in relation to having sex, negatively influence their sexual life during, and even after, treatment (Appleton, 2002).

The results of the studies referred to above confirmthe findings of the current study. On the other hand, according to a study, women’s sexual function has a positive correlation with their male partners’ sexual function; in other words, sexual dysfunctions in women affect their sexual partners (Nelson et al., 2008). Likewise, other studies have found that 20% of husbands in infertile couples, following the diagnosis of infertility or sexual dysfunctions in their wives, experience lower levels of sexual satisfaction (Quintero et al., 2005; Shindel et al., 2008). Theresults of the present study show that the duration and causes of infertility have different effects on sexual burnout in husbands and wives.

The results of the above-mentioned studies are in agreement with the present study. Among the limitations of the study were the stressfulness and noise of the clinical environment and the couples’ anxiety while completing the questionnaires. In view of the importance of sexual issues and the impact of women’s sexual dysfunctions on their husbands, holistic treatments are recommended for infertile couples, including counseling, education, and attention to sexual dysfunctions.

## 5. Conclusion

The results of the study show that couple burnout is significantly higher in infertile women than their husbands. Due to the importance of child-bearing in the Iranian culture, infertile women may think that their inability to conceive a child makes them less attractive to their sexual partners, which idea in turn reduces their sexual desire and makes them sensitive to sexual matters. The eventual result is couple burnout and a decrease in their quality of life. It appears that infertility is not simply a medical problem, and psychotherapeutic interventions need to be introduced into the treatment process to provide couples with psychological support.
